# How Water and Ion Mobility Affect the NMR Fingerprints of the Hydrated JBW Zeolite: A Combined Computational‐Experimental Investigation

**DOI:** 10.1002/chem.202202621

**Published:** 2022-10-10

**Authors:** Siebe Vanlommel, Alexander E. J. Hoffman, Sam Smet, Sambhu Radhakrishnan, Karel Asselman, C. Vinod Chandran, Eric Breynaert, Christine E. A. Kirschhock, Johan A. Martens, Veronique Van Speybroeck

**Affiliations:** ^1^ Center for Molecular Modeling (CMM) Ghent University Technologiepark 46 9052 Zwijnaarde Belgium; ^2^ Center for Surface Chemistry and Catalysis KU Leuven Celestijnenlaan 200 f, PO Box 2461 3001 Leuven Belgium; ^3^ NMR-Xray platform for Convergence Research (NMRCoRe) KU Leuven Celestijnenlaan 200 f, PO Box 2461 3001 Leuven Belgium; ^4^ National High Magnetic Field Laboratory 1800 E. Paul Dirac Dr. Tallahassee FL 32310 United States

**Keywords:** ab initio calculations, aluminium distribution, NMR spectroscopy, water mobility, zeolites

## Abstract

An important aspect within zeolite synthesis is to make fully tunable framework materials with controlled aluminium distribution. A major challenge in characterising these zeolites at operating conditions is the presence of water. In this work, we investigate the effect of hydration on the ^27^Al NMR parameters of the ultracrystalline K,Na‐compensated aluminosilicate JBW zeolite using experimental and computational techniques. The JBW framework, with Si/Al ratio of 1, is an ideal benchmark system as a stepping stone towards more complicated zeolites. The presence and mobility of water and extraframework species directly affect NMR fingerprints. Excellent agreement between theoretical and experimental spectra is obtained provided dynamic methods are employed with hydrated structural models. This work shows how NMR is instrumental in characterising aluminium distributions in zeolites at operating conditions.

## Graphical abstract

Discovering the aluminium location is a challenging endeavour in zeolites, as overlapping resonances complicate spectral assignment. Combining theoretical and experimental NMR is indispensable in spectral interpretation while the inclusion of configurationally diverse water molecules is essential.

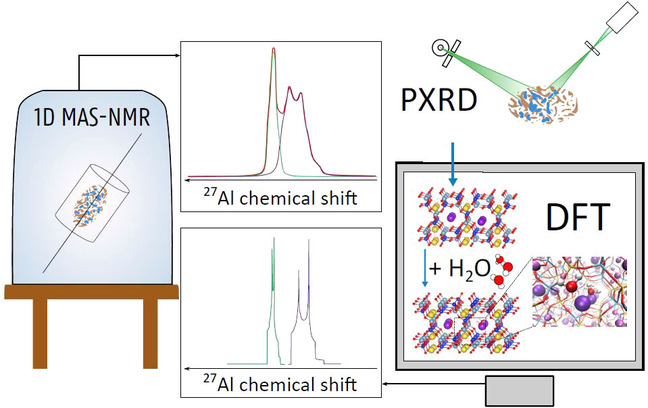

## Introduction

Zeolites are of undeniable importance in many current industrial applications, such as separation and catalysis, and will remain vital to convert non‐fossil based feedstocks and develop sustainable chemical processes.[^1^, ^2^, ^3^] Their excellent large‐scale applicability can be attributed to their hydrothermal stability and attractive nanostructural architecture. Being composed of tetrahedrally coordinated silicon and aluminium sites forming a network of pores and channels, zeolites form a class of shape and size selective materials fit for the diffusion and reactivity of specific molecules.[^3^, ^4^] Catalytic activity in aluminosilicate zeolites stems from the isomorphic substitution of silicon for aluminium and the presence of counterions to retain the overall electroneutrality of the framework. These positive countercharges can be protons bound to neighbouring oxygen sites, resulting in Brønsted acid sites, or extra‐framework cations that reside near the aluminium sites. The catalytic activity of active sites and the interactions with guest species may substantially differ depending on the specific aluminium location.[^5^, ^6^, ^7^, ^8^, ^9^, ^10^, ^11^, ^12^, ^13^, ^14^] As such, the aluminium distribution is important for the final functional behaviour of the zeolite and nowadays specific synthesis techniques have been developed that allow directing the aluminium distribution through templates or structure directing agents,[^8^, ^10^, ^14^, ^15^, ^16^, ^17^] thereby paving the way for control of the active site distribution and the catalytic activity. To include the aluminium distribution in the design process, advanced techniques are necessary to characterize the local structure of the aluminium sites at operating conditions. This requires characterisation techniques that are sensitive on ranges of a few Ångstrom. In this respect, NMR spectroscopy is an ideal candidate because it is able to probe the local environment of atoms through manipulation of nuclear spin populations.[^18^, ^19^, ^20^, ^21^, ^22^] Experimental NMR is ideally complemented by computational techniques in order to provide an unambiguous assignment of the NMR spectrum.[^23^, ^24^] Previous work by Dib *et al*. has shown that combined computational/experimental NMR spectroscopy enables the assignment of aluminium to a specific set of symmetry inequivalent tetrahedral sites in ZSM‐5.^[25]^ However, as the aluminium content is increased, the local geometry of aluminium sites is distorted, and aluminium sites can no longer be considered isolated,^[26]^ which complicates distinguishing tetrahedrally coordinated aluminium sites. Furthermore, as will be shown in this paper, the effect of hydration cannot be neglected: as water molecules interact with countercharges and framework oxygens, both experimental spectra and computational NMR properties change with the amount of water in the system. In order to calculate reliable NMR properties that are comparable to experimental spectra, one must therefore keep in mind that three factors mainly influence the NMR properties: (i) crystallographic symmetry of the T‐site, (ii) possible proximity and distribution of Al sites (which is intrinsically related to the Si/Al ratio) and (iii) presence of water and extraframework species and their interactions with the framework.

**Figure 1:**
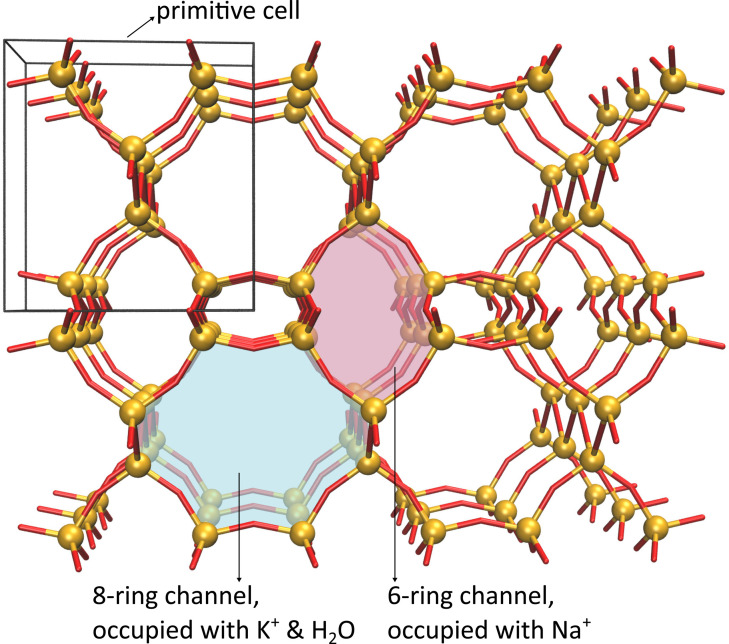
Topology of the JBW zeolite showing the connectivity of the T‐sites that make up the framework.

In this work, we study the JBW−Na,K zeolite with a Si/Al ratio of 1. The crystallographic analysis of this sample by X‐ray diffraction (XRD) revealed exceptional levels of crystallinity and order. The framework consists of one‐dimensional eight‐ring (8R) channels separated by dense anhydrous layers of six‐ring (6R) pores, as is shown in Figure 1. The structure is stabilised by the presence of two types of cations: sodium ions are located in the dense anhydrous layers, whereas hydrated potassium ions reside in the 8R channels. The locations of aluminium sites are known because at Si/Al=1 there is only one possible distribution of aluminium that satisfies Löwenstein's rule of aluminium avoidance.^[27]^ Moreover, there is no change in aluminium distribution possible, as each aluminium atom is restricted to one specific site. In zeolites at a higher Si/Al ratio, it is known that the presence of water assists the breaking of framework Al−O and Si−O bonds,[^28^, ^29^] whereby it is possible that aluminium is removed from the framework. In the JBW system, we have not found evidence of such aluminium mobility. Additionally, ^1^H NMR shows no evidence of the presence of Al‐OH or Si‐OH defects. The JBW zeolite is therefore perfectly suited to unambiguously study the effect of water and crystallographic T‐site symmetry on the NMR signals. The synthesis of the JBW sample was described in a previous paper,^[30]^ in which results from XRD measurements, ^27^Al, ^29^Si, ^39^K and ^23^Na NMR and Rietveld refinements may be found. The aim of the study at hand is to benchmark a combined computational and experimental characterisation protocol for aluminium in zeolites using NMR spectroscopy at operating conditions, critically assessing the role of water. From a computational point of view, both static and dynamic models are employed to compare computational NMR parameters with *operando* spectroscopic 1D MAS NMR (magic angle spinning NMR) signals of ^27^Al and ^29^Si nuclei in JBW at various water loadings. The Si/Al ratio of 1 together with the high level of crystallinity result in very well resolved individual quadrupolar Pake patterns (one per crystallographic site), which is rare in zeolites. The ^27^Al spectrum was measured at different levels of hydration of the material, which provides unique insight into the effect of water on the aluminium NMR parameters. Through comparison of the experimental and computational chemical shift and quadrupolar coupling constant (QCC), the need for *operando* modelling techniques that explicitly take into account the presence of water is shown. The insights obtained in this study are important for future efforts in tuning the aluminium distributions in zeolites at operating conditions.

## Methods

To assess the influence of water on the NMR spectra, the hydrated as‐synthesised JBW sample was dried and rehydrated to the point of saturation, while collecting ^27^Al MAS NMR spectra at specific water loadings along the procedure. All experimental NMR measurements with various hydration levels were carried out following the methodology as detailed in the SI (section S1). ^29^Si, ^23^Na and ^39^K NMR spectra were recorded for the as‐synthesised system and the fully anhydrous system. XRD measurements were employed to obtain the space group, atomic positions and unit cell parameters of the as‐synthesised and dehydrated systems with the purpose of obtaining a starting point to build structural models for the computations. Any reported hydration levels were measured using ^1^H NMR.^[31]^ We note that in ^1^H NMR hydration measurements the experimental water content at the saturation point is underestimated due to chemical exchange between free water and adsorbed water.

The computational structural models were built by taking the atomic positions from the XRD experiments and subsequently loading the structures with different amounts of water. This resulted in a total of six systems of which one triclinic anhydrous cell and five orthorhombic systems with water loading ranging from 0.0 H_2_O/Al (anhydrous) to 0.33 H_2_O/Al (4 water molecules per unit cell). The saturation point of water was determined through grand canonical Monte Carlo, the details of which may be found in the SI (section S2). The computational point of saturation amounts to one water molecule per potassium site, which is in agreement with the XRD measurement performed on our as‐made JBW sample where potassium and water molecules alternate inside the 8‐ring channel as well as with older XRD measurements on the Na,K‐JBW system.^[32]^

The six resulting structural models were then used as input for a static model and for a dynamic model. In the static model, the atomic positions and cell parameters are first optimized using density functional theory (DFT) at the PBE−D3 level of theory. The optimized structures are subsequently used to calculate the NMR parameters through linear response. The dynamic model, on the other hand, uses the XRD structures as input for first‐principles molecular dynamics runs at 300 K. Then a set of snapshots from the trajectories is selected to obtain time‐averaged NMR properties for each atom in the structures. Details of both static and dynamic workflows are given in the SI (section S2). The conversion of calculated chemical shieldings to chemical shifts which can be compared to experiment is detailed in the SI (section S3).

## Results and Discussion

### Experimental characterisation

**Figure 2:**
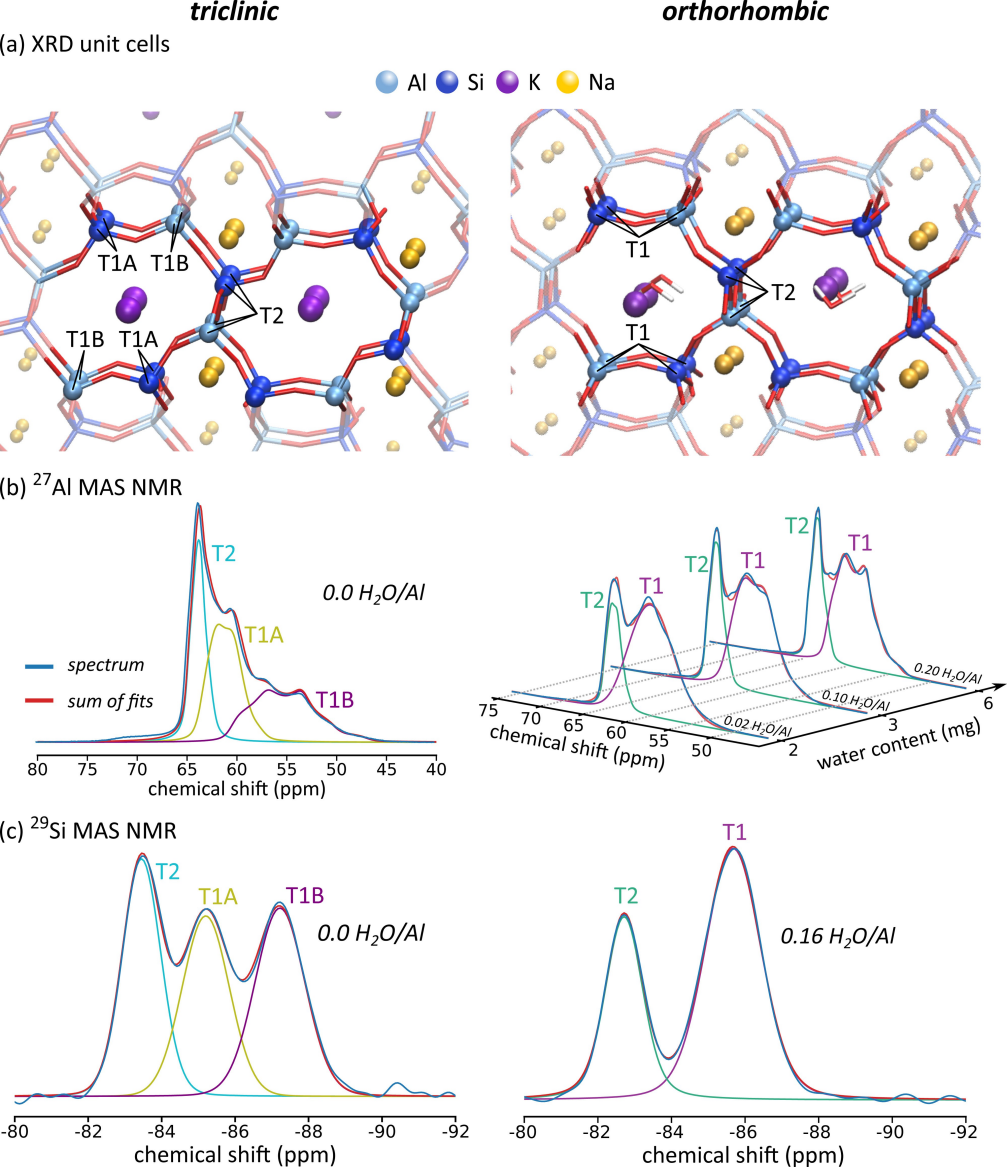
Experimental characterisation of the triclinic and orthorhombic JBW samples used in this work. (a) XRD unit cells with designation of crystallographic T‐sites. Locations of potassium and water are indicative and can vary throughout the 8‐ring channel. (b) ^27^Al MAS NMR spectra at various water contents. (c) ^29^Si MAS NMR spectra.

The space group of the JBW zeolite depends on the degree to which the sample is dehydrated. The as‐made JBW sample has the *Pmn*2_1_ space group showing an orthorhombic unit cell. If the system is dried for 16 hours under vacuum (1 mbar) at a temperature of 200 °C to the point where no NMR‐measurable amount of water is present, the symmetry of the system breaks and it resorts in the $P\bar{1}$ space group with a triclinic unit cell. This breaking of symmetry affects the number of distinct T‐sites in the system, with the orthorhombic system having two distinct T‐sites occurring in a ratio of 2 : 1 (T1:T2), whereas the triclinic system has three T‐sites in ratios of 1 : 1 : 1 (T1A:T1B:T2) (see Figure 2). If the as‐made system is dried for 18 hours under milder conditions (1 mbar, 60 °C), the orthorhombic symmetry is retained and no substantial changes in the XRD pattern are detected. The ^27^Al and ^29^Si MAS NMR spectra of the orthorhombic and triclinic systems are shown in Figure 2, along with their respective XRD unit cells. Numerical results of fitting the NMR spectra and the XRD cell parameters may be found in Table 1. Additional ^23^Na and ^39^K NMR data may be found in the SI (section S7).

**Table 1:** XRD cell parameters, numerical NMR data of experimental ^27^Al NMR spectra and the assignment of the NMR lineshapes to T‐sites for the triclinic and orthorhombic systems.

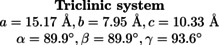										
Water	Adsorbed water	T1A			T1B			T2		
in rotor	in zeolite	*δ_iso_*	*C_Q_*	*η_Q_*	*δ_iso_*	*C_Q_*	*η_Q_*	*δ_iso_*	*C_Q_*	*η_Q_*
(mg H_2_O)	(H_2_O/Al)	(ppm)	(MHz)	(−)	(ppm)	(MHz)	(−)	(ppm)	(MHz)	(−)
0	0.00	63.83	2.66	0.41	61.08	3.95	0.46	65.01	1.73	0.66


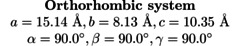										
	Water	Adsorbed water		T1			T2			
	in rotor	in zeolite		*δ_iso_*	*C_Q_*	*η_Q_*	*δ_iso_*	*C_Q_*	*η_Q_*	
	(mg H_2_O)	(H_2_O/Al)		(ppm)	(MHz)	(−)	(ppm)	(MHz)	(−)	
	2	0.02		62.51	3.19	0.53	64.42	1.90	0.50	
	3	0.10		61.88	3.17	0.39	64.01	1.85	0.62	
	6	0.20		61.76	3.16	0.40	63.73	1.80	0.69	
	12	0.21		61.76	3.17	0.40	63.72	1.79	0.70	
	24	0.20		61.76	3.16	0.40	63.73	1.80	0.69	

**Figure 3:**
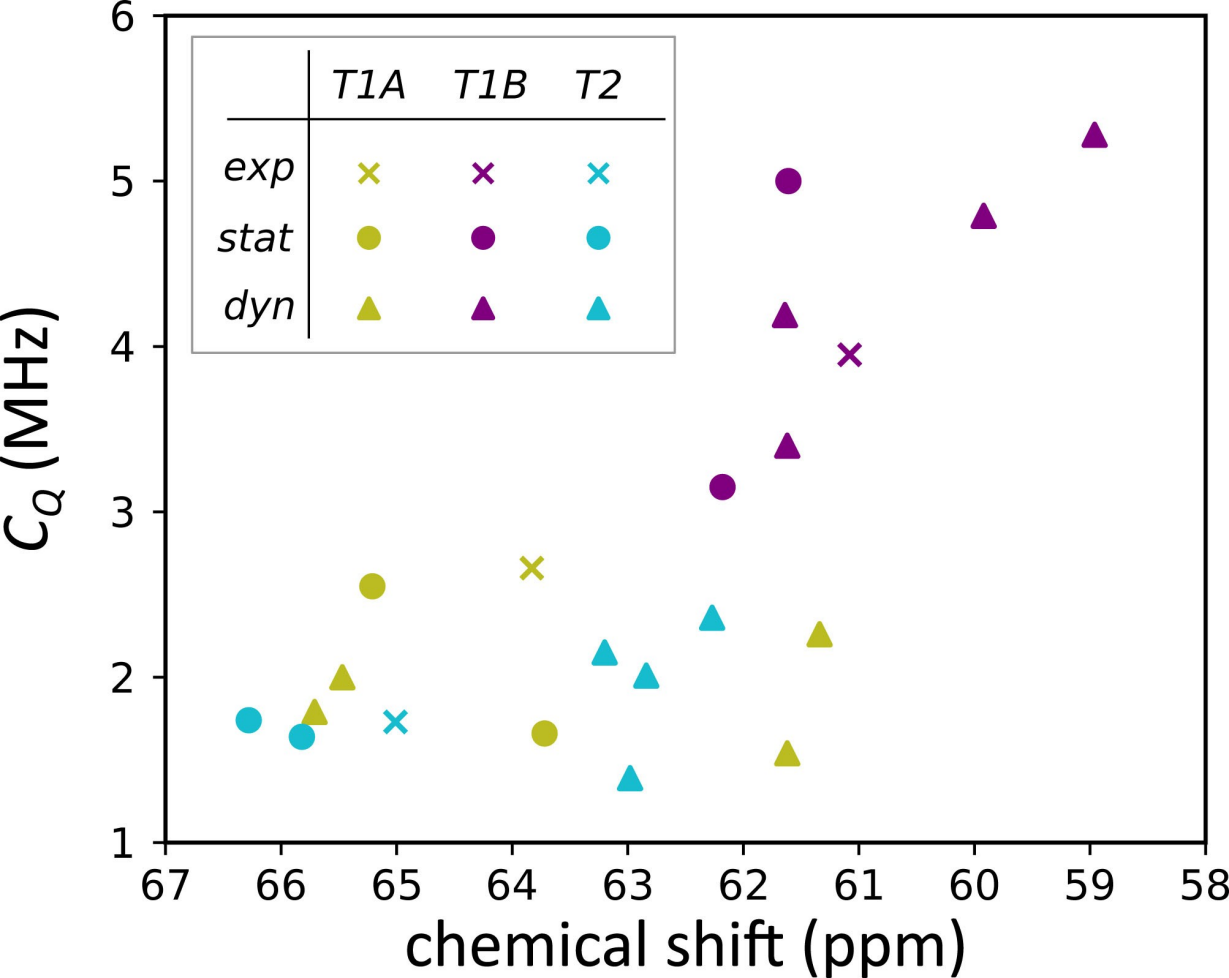
Computational and experimental chemical shift and quadrupolar coupling constant for the triclinic system. The inset table denotes the assignment of the resonances to distinct T‐sites, where *exp*, *stat* and *dyn* respectively denote experimental, static model and dynamic model. Resonances of the static model overlap, as each resonance occurs pairwise.

The strongest influence of water on the ^27^Al NMR parameters is found at low water loadings. Beyond 6 mg H_2_O, the influence of adding additional water to the rotor on the aluminium NMR parameters is negligible, as can be seen in Table 1. This indicates that no significant amount of extra water is adsorbed in the zeolite beyond this point, which is consistent with the ^1^H NMR measurements. The ^27^Al NMR spectra for the orthorhombic system as shown in Figure 2 can be fit using two quadrupolar lineshapes with the integrals under the curves amounting to 67 % and 33 % of the total spectrum. Similarly, the ^29^Si NMR spectrum in Figure 2 contains two sharply defined gaussian lineshapes, again amounting to 67 % and 33 % of the total spectrum. Based on the relative contributions to the spectrum, the lineshape contributing 67 % is assigned to aluminium and silicon sites located at T1 positions, and the lineshape contributing 33 % is assigned to T2 positions (Table 1). We note that the driest spectrum, where 2 mg of water is present, shows a slightly larger fitting error (the root‐mean‐squared deviation of the fitted spectrum from the experimental spectrum is 4 % higher than for the spectrum at 6 mg). This can be explained by the presence of a slight distribution of local geometries in the case where little water is present, whereas these minor differences are averaged out if more water is present. In the triclinic system, three lineshapes are needed to fit the ^27^Al and ^29^Si NMR spectra properly. Based on the assignment in the orthorhombic case, the lineshape with the highest chemical shift (65.01 ppm) is assigned to aluminium residing in T2 sites. Purely based on experimental data, it is impossible to assign the other two lineshapes to T1A and T1B sites. The assignment of the triclinic case in Table 1 is therefore based on the comparison with computational chemical shifts as derived in the next section: the lineshape at 63.83 ppm is assigned to T1A sites and that at 61.08 ppm to T1B sites (see Figure 3).

### Computational NMR data

Figure 3 shows the comparison of the ^27^Al chemical shift and QCC between the static model, dynamic model and the experimental fitting data for the anhydrous triclinic cell. The quadrupolar asymmetry parameter and ^29^Si chemical shift may be found in the SI (section S4). Based on the data in Figure 3, the following observations can be made. Neither the static nor the dynamic model produces three distinct resonances, contrary to what could be expected based on the presence of three symmetrically distinct T‐sites (see Figure 2). Atoms residing in the T1A, T1B or T2 sites show different NMR resonances, and there is no immediate distinction between the three symmetry labels. The T1B sites are slightly more separated from the T1A and T2 sites, which is consistent with the experimental resonance that lies further from the other two resonances. There is a significant overlap in both chemical shift and QCC for atoms residing in T1A and T2 sites. The fact that there is a large spread on the values for both the dynamic and static models compared to the single value per crystallographic site obtained in the experiment, can be explained by considering the position and mobility of potassium which may have more configurational freedom in the 8R channels depending on the amount of water present in the structure. We will return to this point in the section ‘On the role of mobility’. Additionally in the dynamic model, the limited time scale of molecular dynamics simulations (100 ps) compared to the experimental time scale induces a spread as well (see section S9 of the SI for convergence of NMR properties with respect to simulation time).

**Figure 4:**
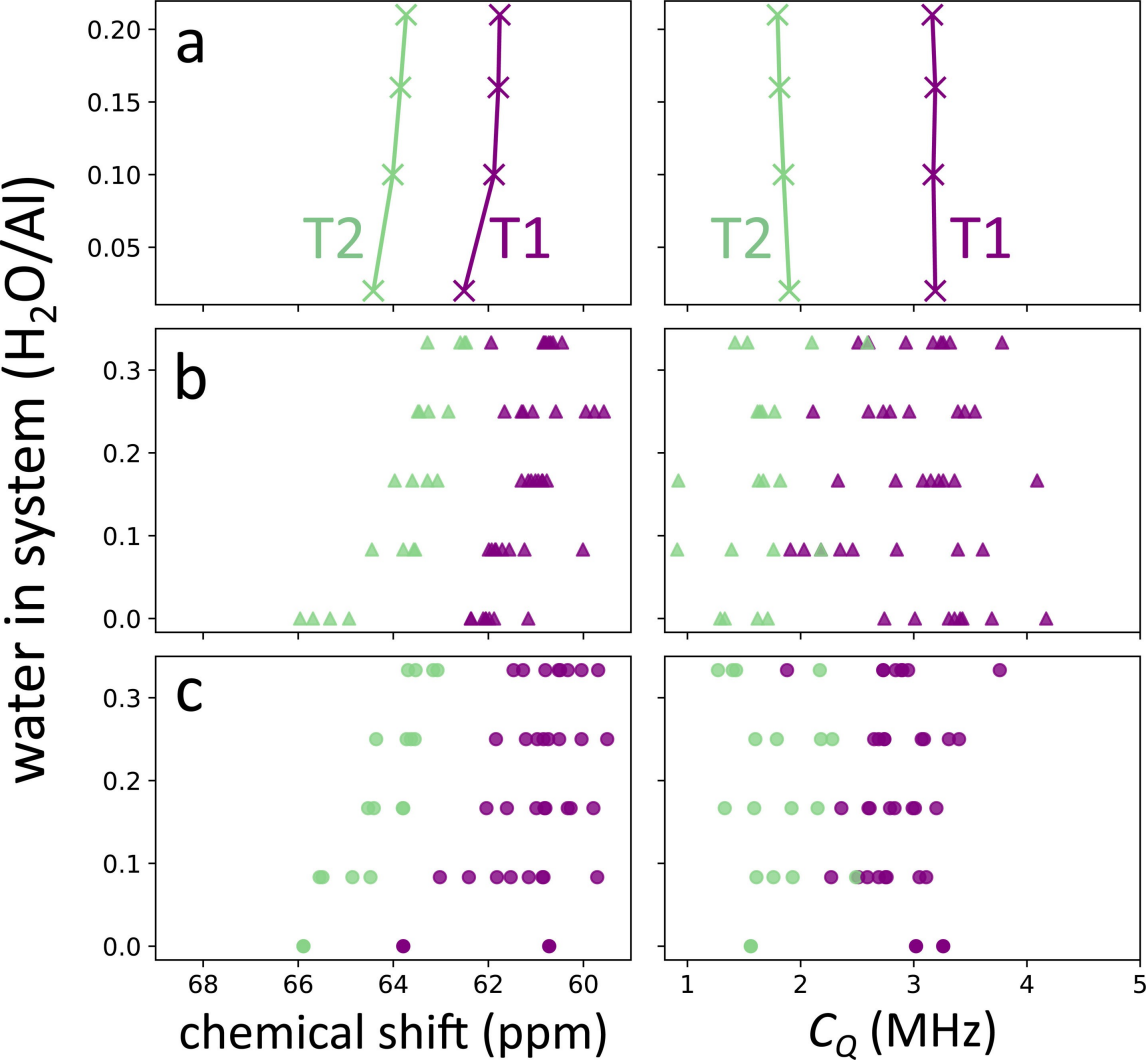
Experimental (panel a), dynamic model (panel b) and static model (panel c) chemical shift and quadrupolar coupling constant for the orthorhombic system plotted against the water loading.

Before considering the role of potassium, we turn our attention to the orthorhombic modelling results. The ^27^Al chemical shift and QCC for all measured and simulated hydration levels are shown in Figure 4. The top plots (panel a) show the experimentally fitted NMR parameters, where two quadrupolar lineshapes were used, assigned to T1 and T2 sites. The middle (panel b) and lower (panel c) plots respectively show the dynamic and static chemical shifts and QCC, coloured according to whether the specific aluminium atom resides in a T1 or T2 position. The quadrupolar asymmetry parameter and ^29^Si chemical shift data may be found in the SI (section S4). Additionally, we have produced a plot that shows the correlation between the average T−O−T angle and the chemical shift, which may be found in the SI (section S5). Note that for each hydration level, 8 T1 and 4 T2 data points are plotted, however, some of them overlap. The computational ^27^Al chemical shift and QCC show a clear splitting between resonances originating from T1 and T2 sites. Moreover, it is clear that both the static and dynamic model can reproduce the trend of decreasing chemical shift upon increasing water loading. For the QCC, there is no obvious trend when changing the hydration level. While the individual resonances of the aluminium sites change upon the addition of water, the overall distribution of values does not show a systematic change. This is also the case for the experimentally fitted values as these only change by 0.1 MHz overall, which is small compared to the spread on computational *C_Q_* values. An important phenomenon to notice is that there are three distinct resonances in the static model for the anhydrous case (Figure 4, panel c), whereas the orthorhombic framework only contains two distinct T‐sites. As was the case for the spread on NMR parameters in the triclinic cell, this too is affected by the choice of potassium location. However, the important difference between the two systems is that the dynamic model only shows two distinct resonances in the anhydrous orthorhombic case, as evidenced by the fact that the chemical shifts are split into and centered around two contributions near 66 and 62 ppm (see column of chemical shift in Figure 4, panel b). It is also worth noting that the anhydrous dynamic model produces an average chemical shift that is close to the one produced by the static model. This is especially clear for the resonances of the T2 site, while for the T1 sites the dynamic anhydrous shifts lie close to the average of the two static anhydrous values. This indicates that the motion of atoms around their equilibrium positions does not change the NMR parameters a lot. However, the introduction of water into the system significantly affects the NMR response of individual sites.

Overall, while the computational NMR chemical shifts are clearly split into T1 and T2 sites in the orthorhombic case, not all sites show the same NMR resonance. The main reason for the inequivalence in the hydrated cases, next to the effect of potassium, is the absence of water dynamics. In the static model, each aluminium atom sees one particular water configuration. In the dynamic model, water positions may vary, however, limited configurational averaging still renders the individual resonances inequivalent. The reason for this is twofold: (a) no diffusion of water is present in the model system and (b) the time scale of the dynamic simulations is too short to attain the same configurational diversity as in the experimental system. In the real system, more configurational diversity is present so that over time all individual atoms with the same T‐label, on average, see the same water configurations around them, which then translates into a single NMR resonance. While these long time scales are not accessible in the dynamic model (the forces and interactions are calculated at the DFT level), the necessary configurational diversity of water can be mimicked by further averaging over T‐sites within one crystallographic label, as will be explained in the section Bridging experiment and theory.

### On the role of mobility

**Figure 5:**
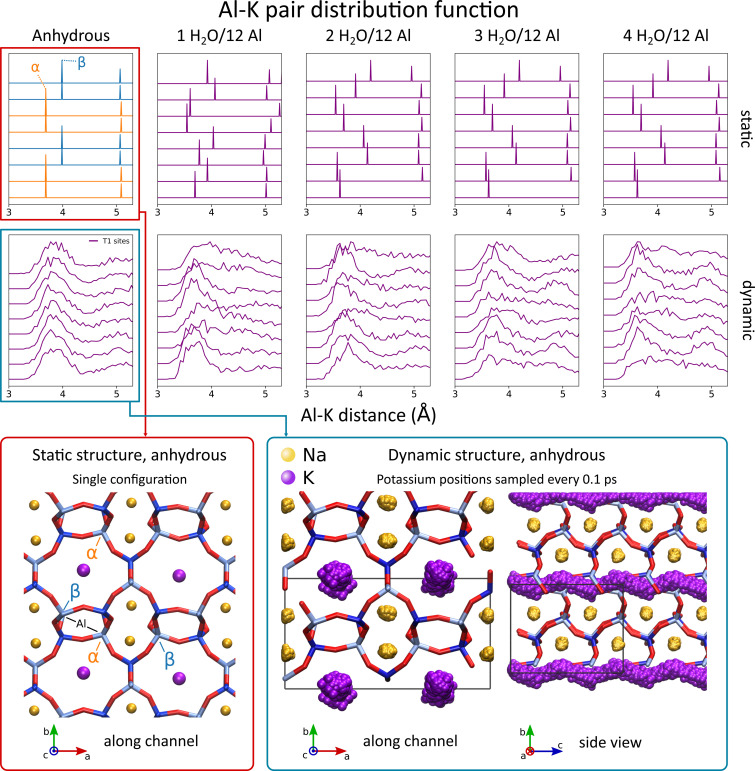
Static (top) and dynamic (bottom) Al−K pair distribution functions for the orthorhombic system show that in the dynamic simulations, potassium is free to move between different possible locations. The static structure is shown in which T1 sites with different distances to closest potassium ions are indicated (*α, β*), as well as the location of potassium in the dynamic MD run, showing the large mobility of potassium that cannot be captured by the static model.

It was already noted that both the static and the dynamic model fail to account for a realistic dynamic water loading. However, the ^27^Al chemical shift of the individual sites in the dynamic model of the orthorhombic system (Figure 4 panel b) does not show the clear splitting of the T1 sites into two resonances, as it does in the static model, which points to an additional important difference. In the static case, the T1 sites after optimisation are split into sites with closest potassium ion at a distance of 3.6 Å and sites where this distance is 3.9 Å (indicated *α* and *β* in Figure 5, respectively). However, investigation of the dynamic trajectories in the orthorhombic system reveals that potassium ions are largely mobile in the anhydrous case and can even diffuse through the channel of the system (see Figure 5). The result is that the distribution of distances between the closest potassium ion and T1 sites is not sharply centered around 3.6 Å or 3.9 Å, but rather all T1 sites show a comparable distribution in the anhydrous Al−K pair distribution functions, as is clear from Figure 5 in the first column. With no water present, potassium ions are free to occupy all possible potassium sites, which is consistent with the hypothesis by Healey *et al*. that potassium ions occupy sites that lead to plausible interaction distances between water oxygens and the cations.^[32]^ The spread on chemical shift in the orthorhombic dynamic model is then a consequence of slight diversity in average local geometries and due to limited sampling of water configurations. In case water is present, the potassium ions are still sufficiently mobile to remove the clear distinction between the *α* and *β* sites, as evidenced in Figure 5 from the second to the last column. Only if a dynamic model is used, the differences introduced due to potassium location are averaged out and overall we recover two inequivalent sites, consistent with the two lineshapes in the experimental NMR spectrum.

Finally, we return once more to comment on the mobility of potassium in the triclinic case in which there are two distinct T1 sites, labelled T1A and T1B. The static and dynamic Al−K pair distribution functions for the triclinic system may be found in the SI (section S6). The data indicates that our model even predicts further distinction within the T1A and T1B labels, as within these labels there exist sites with distinct Al−K distribution functions. Furthermore, no potassium diffusion through the channel is observed in the trajectory of the system. We therefore conclude that mobility is hindered in the triclinic cell, leading to discrepancies in NMR parameters between models using a single K distribution and experiment. Interestingly enough, this is consistent with previous observations in the JBW system that upon rehydrating the anhydrous triclinic cell, the orthorhombic cell is not retrieved,^[30]^ also pointing to inhibited mobility of potassium ions and water molecules.

### Bridging experiment and theory

**Figure 6:**
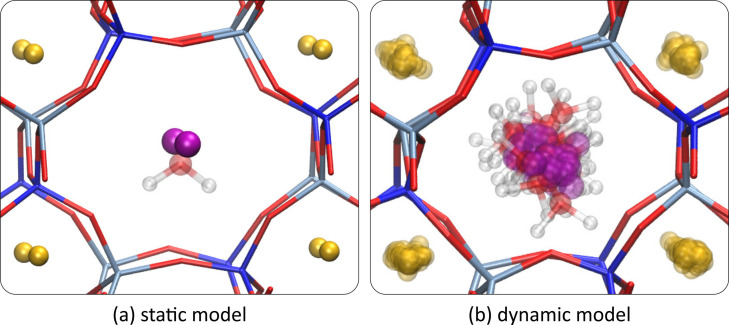
View along the hydrated 8‐ring channel in the case of a single water molecule in the orthorhombic unit cell for the (a) static and (b) dynamic models. Only dynamics of non‐framework species are shown for the dynamic model. Treating the T‐sites within the same T‐site label as being equivalent, provides us with representative average NMR parameters comparable to the real case where water is mobile.

In this section we aim to obtain a direct comparison between the experimental ^27^Al NMR spectrum and model NMR parameters derived from theory for the orthorhombic case. As argued before, the T1 (respectively T2) sites at each specific water loading are nearly equivalent in the orthorhombic dynamic models. Therefore, it is appropriate to further average over T‐sites within the T1 (respectively T2) label to obtain a representative computational T1 (T2) resonance which can be compared to the experimentally fitted T1 (T2) data. The reasoning behind this, is that in the real system, the NMR parameters are measured on much larger time and length scales than in the computational models. In our dynamic model no diffusion of water molecules past the potassium ions in the channel is observed on the time scale accessible by these dynamic models (around 100 ps), as these are calculated at the DFT level. Water does diffuse in the experimental system during measurement otherwise no substantial change in NMR spectra would be observed upon hydration. The mobility of water and potassium species and a comparison to the static single‐configuration case is shown in Figure 6. Averaging over different T‐sites with the T1 (T2) label is a representative model that includes more configurational diversity. In the hydrated orthorhombic system, multiple orientations and locations of both water molecules and potassium ions are taken into account in this way. In the anhydrous triclinic case, diffusion of potassium is hindered and we are unable to obtain a representative average using a model with only one potassium distribution, therefore we do not consider it any further. Similarly, averaging over different T‐sites cannot be justified in the static model because the lack of mobility of potassium leads to additionally induced inequivalence of T‐sites. Given previous arguments, static results are not further considered to directly compare theoretical and experimental NMR spectra. In averaging, one must take care of considering symmetry transformations that must be applied before deriving average NMR properties. Details on this procedure may be found in the SI (section S10).

The result of this averaging procedure at each water loading for the dynamic model is shown in Table 2. The most relevant parameter to compare the average values with the experimental chemical shift, is the difference in ppm between the two sites, denoted Δ(T2‐T1). It is clear that the difference lies close to the experimental values, except in the anhydrous model, where the discrepancy is substantially larger. This overestimation of the chemical shift difference can be explained by the following facts: (i) the experimental orthorhombic system is not fully anhydrous, while the model system is and (ii) the cell parameters of the anhydrous run are fixed to be the parameters of the XRD experiment whereas this was not the case for the other water loadings, because in the anhydrous case the triclinic cell is energetically more stable than the orthorhombic cell. Further discussion on this may be found in the SI (section S8). The difference in chemical shift Δ(T2‐T1) is reproduced best at saturation, with an experimental Δ of 1.97 ppm and a model Δ of 1.85 ppm. The QCC is reproduced with a good accuracy at saturation, with model values of 1.55 MHz and 3.06 MHz, underestimating the experimental values of 1.80 MHz and 3.16 MHz by 0.25 MHz and 0.10 MHz, respectively. The asymmetry parameters of 0.69 and 0.30 for the average model compare well to the experimental values at saturation of 0.69 and 0.40. The model approaches the experimental values most closely at the lowest water loading (where the two T‐sites show a very similar asymmetry parameter) and at saturation (where they are separated). In general, the quadrupolar parameters are in good agreement in the driest possible case, and in the saturated case. However, the chemical shift difference is not reproduced with satisfactory precision in the anhydrous model, which means that overall the model performs best in reproducing the experimental values at saturation. The fact that individual resonances of all NMR parameters lie in a broad range of values, while the averages per label produces values that are comparable to experimental data can be seen as proof that averaging over many water configurations is necessary to obtain reliable NMR parameters.

**Table 2:** Comparison of experimental data to average ^27^Al NMR T1/T2 resonances calculated using data of the dynamic model. The best agreement between experimental and model parameters is obtained at saturation (top row).

		**Experiment**			
Hydration	*δ_iso_* (ppm)	*C_Q_* (MHz)		*η_Q_* (−)	
(H_2_O/Al)	Δ (T2‐T1)	T2	T1	T2	T1
>0.21	1.97	1.80	3.16	0.69	0.40
0.16	2.06	1.81	3.19	0.69	0.39
0.10	2.13	1.85	3.17	0.62	0.39
0.02	1.91	1.90	3.19	0.50	0.53

		**Dynamic, average**			
Hydration	*δ_iso_* (ppm)	*C_Q_* (MHz)		*η_Q_* (−)	
(H_2_O/Al)	Δ (T2‐T1)	T2	T1	T2	T1
0.33	1.85	1.55	3.06	0.69	0.30
0.25	2.61	1.63	2.86	0.58	0.34
0.17	2.48	1.18	3.10	1.00	0.37
0.08	2.32	1.29	2.51	0.82	0.45
0.00	3.48	1.37	3.27	0.63	0.57

**Figure 7:**
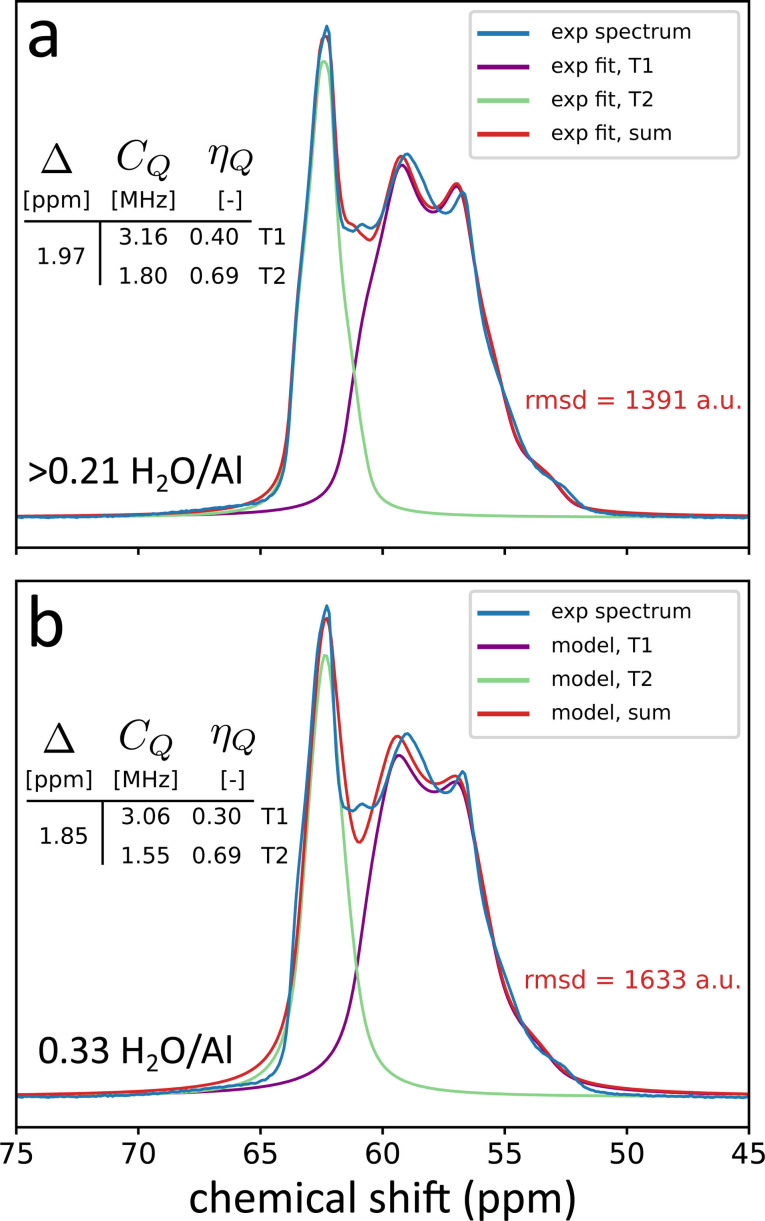
Comparison of experimental fit to the dynamic average model as detailed in section Bridging experiment and theory and comparison of experimental spectrum to the experimental fitting data. The spectrum simulated using the average dynamic model parameters (bottom, panel b) is compared to the fit of the experimental spectrum (top, panel a).

The simulated model spectrum at saturation, if artificially broadened as is routinely done to fit the experimental spectra, compares well with the experimental spectrum. This can be seen in Figure 7. The spectrum was simulated by using the parameters as determined in the average dynamic model with two species (T1, T2). The only variables that were optimized to minimize the RMSD between the simulated and experimental spectrum, were the area under the curve, the width of the Lorentzian broadening and a constant that can translate the simulated spectrum as a whole without altering Δ(T2-T1) . The dynamic model spectrum was simulated using a fixed ratio of T1 to T2 lineshape contributions of two to one. Following this procedure, it is clear that the dynamic computational model can reproduce the experimental spectrum with great accuracy with an overall root‐mean‐square deviation (rmsd) that is comparable to the experimental fits using two lineshapes.

## Conclusion and outlook

In this work, we have investigated the JBW zeolite with a Si/Al ratio of 1. Owing to its fully defined aluminium distribution at Si/Al=1, this zeolite serves as a good benchmark system to determine the influence of water on the NMR spectra and to set up a protocol for reliably determining computational NMR properties. Experimental ^27^Al 1D MAS NMR measurements were performed at different hydration levels going from as dry as possible to fully hydrated, revealing the dependency of the NMR parameters on the water content. The chemical shift is the parameter which is most affected by the specific water content, showing a steep decrease upon water loading. Both static and dynamic models were employed to model this behaviour, explicitly taking into account the presence of water. While both models are able to reproduce the trend in chemical shift, the dynamic model gave the best agreement with the experiment as in this approach the mobility of the potassium species and water molecules is taken into account. Furthermore, the spread on individual resonances of the atoms is large in both models. We have proposed a way to enhance the comparison to experiment, by averaging over sites in the model that are located at crystallographically equivalent positions. This is especially relevant for systems with very high aluminium content, similar to the system at hand, where many aluminium atoms are located at sites with the same framework symmetry. As such, the experimental ^27^Al NMR spectrum can be reproduced with great accuracy, provided the comparison is done at saturated water content and including many different local water configurations.

The data in this work highlight the importance of explicitly taking into account water in the simulation of zeolitic systems when one is interested in obtaining *operando* NMR parameters, *i. e*. at experimental conditions. Under these conditions dynamic averaging cannot be neglected, as many different water configurations contribute to the experimental signal which cannot properly be taken into account in the static model. The obtained results serve as a protocol on how to include the effect of water and its dynamic rearrangement in computational models that aim to determine the NMR fingerprint of aluminium distributions in zeolites under realistic conditions. To extend the work to other zeolites, systems with symmetrically inequivalent T‐sites at various Si/Al ratios should be considered to further elucidate the impact of the aluminium distribution on the NMR properties. In this respect, computational modelling of the aluminium distribution and NMR spectra is vital, as NMR spectra cannot be deconvoluted with certainty in these cases due to the aluminium atoms being located at a plethora of symmetrically inequivalent sites. Moreover, in zeolites with a Si/Al ratio greater than 1, grouping T‐sites into symmetrically distinct classes is a challenge on its own as the definition of symmetry equivalent sites depends on how many coordination shells are taken into account to produce the symmetry labels. While the computational protocol at hand is in principle applicable to such cases, the grouping of sites can become tedious and it might be better to consider each aluminium site on its own and aim to derive well‐averaged NMR parameters by including as much configurational diversity of water clusters as is computationally possible. If, by some definition of equivalent sites, some atoms can be grouped to represent one equivalent T‐site, one can still apply the averaging protocol. Additionally, there is no reason why the presence of water would not have any influence on the NMR spectrum in different zeolitic systems as well, especially in the case of protic zeolites, where it is expected that water plays an even bigger role as it can change the nature of the acid site.[^33^, ^34^] Finally, for zeolites with Si/Al ratio larger than 1, it is possible that considerable mobility of framework atoms is present due to the water‐assisted breaking of Al−O and Si−O bonds.[^35^, ^36^] This can lead to the formation of extraframework species and therefore to the possibility of a changing aluminium distribution, or to the exchange of framework oxygen atoms. In zeolites where such phenomena occur, one can anticipate that there will be an effect on the NMR spectra as well, since in these processes the local structure is affected. With this paper, by investigating the aspect of water content separately from the aluminium distribution, we have taken a step towards developing a methodology that could provide a complete spectroscopic description of the aluminium distribution in aluminosilicate zeolites based on state of the art computational and experimental methods.

## Supporting Information

The following supporting information is available: methodological details on experimental NMR, overview of computational methodology, information on the conversion of shielding to shift values, additional data for the triclinic and orthorhombic systems (quadrupolar asymmetry parameter and ^29^Si chemical shift), Al−K pair distribution functions for the triclinic system, experimental ^23^Na and ^39^K NMR for the as‐synthesised orthorhombic and the anhydrous triclinic systems, detailed analysis of unit cell symmetry using molecular dynamics, convergence tests for the DFT‐GIPAW method with respect to computional settings and number of snapshots, space group transformations used when averaging over T‐sites. For an overview of which structural models and input files are available, we refer the reader to the SI (section S11).

## Conflict of interest

The authors declare no conflict of interest.

1

## Supporting information

As a service to our authors and readers, this journal provides supporting information supplied by the authors. Such materials are peer reviewed and may be re‐organized for online delivery, but are not copy‐edited or typeset. Technical support issues arising from supporting information (other than missing files) should be addressed to the authors.

Supporting Information

Supporting Information

Supporting Information

Supporting Information

Supporting Information

## Data Availability

The data that support the findings of this study are available in the supplementary material of this article.
